# Hearing Loss in Adult Survivors of Childhood Cancer Treated with Radiotherapy

**DOI:** 10.3390/children5050059

**Published:** 2018-05-04

**Authors:** Amber Khan, Amy Budnick, Dana Barnea, Darren R. Feldman, Kevin C. Oeffinger, Emily S. Tonorezos

**Affiliations:** 1 Southwestern Medical School, University of Texas, 5323 Harry Hines Blvd, Dallas, TX 75390, USA; ankhan92@gmail.com; 2 Memorial Sloan Kettering Adult Long-Term Follow-Up Program, 485 Lexington Ave, 2nd Floor, New York, NY 10017, USA; budnicka@mskcc.org (A.B.); Feldmand@mskcc.org (D.R.F.); 3 Tel Aviv Sourasky Medical Center, Survivorship Clinic, Hematology and Oncology, Weizmann St 6, Tel Aviv 64239, Israel; dana.barnea@gmail.com; 4 Department of Medicine, Weill Cornell Medical College, 525 East, New York, NY 10065, USA; 5 Duke Cancer Institute, 2424 Erwin Drive, Suite 601, Durham, NC 27705, USA; kevin.oeffinger@duke.edu

**Keywords:** radiotherapy, ototoxicity, survivorship, hearing aids, pediatric cancer

## Abstract

The ototoxic effects of radiotherapy have been poorly characterized. We examined adult survivors of childhood cancer who were treated with radiotherapy, which included the head, before the age of 22 years and between 1952 and 2016. Those who received platinum chemotherapy were excluded. Demographic, diagnosis, and treatment outcomes were captured. Audiograms were graded using the Chang and International Society of Paediatric Oncology ototoxicity (SIOP) scales. Among 276 patients with a history of radiation to sites that included the brain, orbit, nasopharynx, and total body irradiation, the median age at treatment was 10.1 years and 59% were male. Of 51 survivors who had post-treatment audiograms, 19 demonstrated severe hearing impairment according to both the Chang and SIOP scales after a median follow-up of 16.6 years. Of those with severe impairment, 10 were using hearing aids. Among the 23 patients with more than one audiogram, five had normal hearing on the first audiogram but hearing loss upon subsequent study. Ototoxic effects of radiotherapy are present in a significant portion of survivors, but impairment may present over time, and our results suggest that many are not being screened. Further, among patients with severe hearing loss, use of hearing aids is not universal. Expansion of access to audiology testing and hearing interventions may be warranted.

## 1. Introduction

While cancer mortality has declined in recent decades, late effects of treatment impact the long-term health and quality of life for cancer survivors [1,2]. Hearing loss is one of the most common adverse effects of treatment, with implications for language acquisition, economic outcomes, mental health, and social development in the survivor population [3]. Ototoxic effects are primarily associated with chemotherapy agents cisplatin and carboplatin, as well as high-dose radiotherapy (RT) to the cochlea. Due to the complex anatomy of the head and neck, RT to fields that involve these sites may also impact the ear and the cochlea [4,5].

Radiotherapy-induced hearing loss can present as conductive, sensorineural or mixed hearing loss [4,5,6]. The majority of prior studies on hearing loss in pediatric cancer survivors consists of populations with a median follow-up time under 10 years or rely on questionnaires and self-report [7,8]. Several studies have shown dose-dependent effects of RT on hearing outcomes; associated risk factors include treatment with aminoglycosides, platinum agents, craniospinal fluid (CSF) shunts, and cranial surgery [4,5,7,8,9,10,11,12]. Recent studies have also examined the independent ototoxic effects of platinum therapy and showed early onset, bilateral high-frequency loss following treatment [13,14,15]. However, few studies examined the ototoxic effects of RT independent of platinum therapy using objective audiometric testing over long-term follow-up [6,9,16]. Furthermore, few studies examined the ototoxic effects of RT on survivors of both central nervous system (CNS) and non-CNS cancers, or described the use of assistive devices [6,7,8,9,12,17]. Therefore, we sought to describe objectively-defined post-treatment hearing loss and patterns of hearing aid use among a clinic-based population of adult survivors of childhood and young adult cancer.

## 2. Methods

### 2.1. Patient Population

The Adult Long-Term Follow-Up (ALTFU) program at Memorial Sloan Kettering Cancer Center provides risk-based care to adult survivors of childhood and young adult cancer. A retrospective chart review was conducted for patients seen in the ALTFU Program between September 2005 and January 2017. Memorial Sloan Kettering Institutional Review Board approval was obtained for this research (#16-1523); because of the retrospective nature, the requirement for consent was waived. Eligibility criteria included at least one clinical visit at ALTFU, cancer diagnosis before 22 years of age, and RT to fields that included the cochlea or ear. Radiotherapy fields included the brain, posterior fossa, craniospinal, nasopharynx, skull, face, orbit, total body irradiation, sinus, and the pineal gland. Exclusion criteria included receipt of platinum chemotherapy. Aminoglycoside and occupational exposures were noted when annotated in the audiology report.

We collected information on degree of hearing impairment and use of hearing aids. The RT field and dose were also abstracted. Consistent with the Children’s Oncology Group guidelines for ototoxicity evaluation, we defined low dose RT as <30 Grey (Gy) and high dose RT as ≥30 Gy [18,19].

### 2.2. Audiologic Methods

Two authors evaluated pure tone audiograms performed post-treatment. Bone conduction thresholds were used to determine the presence of sensorineural hearing loss when patients demonstrated mixed or conductive hearing loss. In audiograms with normal tympanograms, air conduction thresholds were used where bone conduction was unavailable. The audiologic evaluation included audiologic history, air and bone conduction thresholds (250–8000 Hz and 250–4000 Hz respectively), speech audiometry, immittance audiometry, and distortion product otoacoustic emissions when applicable. A mixed or conductive hearing loss was designated by an air/bone gap greater or equal to 15 dB and/or flat tympanograms. When a mixed hearing loss was observed, bone conduction thresholds were measured to evaluate sensorineural loss.

Audiometric data were graded according to both the Chang Ototoxicity Scale (Table 1) and the International Society for Pediatric Oncology (SIOP) Scale (Table 2) [20]. Previous studies have shown that the Chang and SIOP scales agree regarding severe ototoxicity, and the Chang scale has been shown to be predictive of hearing aid need post-treatment at grades of 2b and higher [21]. Chang grade ≥ 2b has been shown to correlate with SIOP grade ≥ 3 when defining severe hearing loss, thus, we define severe hearing impairment as hearing thresholds that fulfill these criteria [1,12]. These ototoxicity scales predict functional hearing loss and high-frequency hearing loss, which are relevant to the initial ototoxic effect in both RT- and platinum therapy-induced hearing loss [4,5,12,22]. All results presented are descriptive, and no statistical testing was performed.

## 3. Results

### 3.1. Patient Characteristics

We found 276 survivors with a history of RT and without a history of platinum-based chemotherapy who were seen in the ALTFU program. Approximately one in five (51/276; 18.5%) underwent post-treatment audiology testing at a median of 3.0 years (range, 0–55 years) after the end of treatment. Patients with follow-up audiograms had a younger median age at diagnosis, greater proportion of CNS tumor diagnoses, more equitable gender distribution, greater proportion of hearing aid use, and greater proportion with a history of cranial surgery, compared to those without audiogram (Table 3). Median follow-up time in survivorship care was 4.3 years (range 0–10.1 years). Most common radiation fields included brain and total body irradiation, followed by orbit and nasopharynx. Median radiation doses for all fields are listed in Table 4.

Among the 51 patients with a history of RT who did not receive platinum chemotherapy but had follow-up audiogram, one had Fanconi’s anemia, one was diagnosed with neurofibromatosis 2 and had associated schwannoma, and one had congenital choleastoma and ear surgery. Aminoglycoside treatment was noted in audiology reports and progress notes for three patients; however, many patients treated for infections and transplant treatment may have also received aminoglycoside treatment.

Among these 51 patients, 19 were found to have severe hearing impairment. Survivors with hearing impairment had a median age at diagnosis of 8.0 years (range, 0–19.2 years), and primarily included leukemia and CNS tumor survivors. Cumulative radiation dose exceeded 30 Gy for 13 patients. However, six patients had RT doses less than 30 Gy, a threshold below which patients are not typically screened for hearing loss. Six patients received RT to more than one field. The cumulative RT dose range was from 1350–10,800 Gy.

### 3.2. Audiology Results

International Society of Paediatric Oncology and Chang ototoxicity grading scales were used to grade 119 post-treatment audiograms. A single audiologist performed 108 audiology evaluations at Memorial Sloan Kettering; 11 audiology evaluations were performed at outside institutions. On first post-treatment audiogram, 27/51 (53%) of patients had hearing within normal limits. Amongst the total of 119 audiograms collected, sensorineural hearing loss was observed on 64 (54%), hearing within normal limits was seen on 29 (24%) of the audiograms, and the remaining showed mixed loss in 15 (13%) and conductive hearing loss in 11 (9%).

Fifteen of 51 patients exhibited hearing thresholds consistent with both Chang grade 2b or above and SIOP grade 3 or above on their first post-treatment audiogram. Within this group, four patients had characteristics noted on their audiology report that may affect auditory outcomes: one developed severe otitis media secondary to treatment; one developed acoustic neuroma after treatment; one received RT for an acoustic neuroma; and one received RT for a pediatric and adult cancer.

Changes in ototoxicity between post-treatment audiograms were evaluated for 23 patients with more than one audiogram, starting a median of two years after treatment (range, 0.3–55 years). Nine of 23 (39%) patients demonstrated severe hearing impairment on their first post-treatment audiogram, while an additional five patients (22%) demonstrated severe hearing impairment upon subsequent testing. Therefore, severe hearing impairment emerged over the course of follow-up for a substantial proportion of patients.

Importantly, of 19 patients with a history of RT and severe hearing impairment, only 10 wear hearing aids. All 10 patients wearing hearing aids also demonstrated high-grade ototoxicity on audiologic evaluation. Reasons for not using hearing aids, in spite of eligibility, were not uniformly noted in the chart but included cost and discomfort.

## 4. Discussion

In this evaluation of adult survivors of childhood and young adult cancer exposed to ototoxic therapy, 19 of 51 patients with a history of RT, but without a history of treatment with platinum-based chemotherapy, were found to have severe hearing impairment. Amongst 19 patients with severe impairment, 15 presented with severe impairment on their first post-treatment audiogram, and four had developed severe impairment by the time of subsequent audiograms. Of those with severe impairment, 10 patients used hearing aids.

Our findings are consistent with those of past studies of survivors of childhood and young adult cancers, suggesting that RT-induced ototoxicity may present several years later than hearing loss secondary to platinum therapy, and that many survivors do not have regular audiograms [4,7,16]. Further, while sensorineural hearing loss is most common, survivors may also have components of conductive hearing loss due to exposure of the outer, middle, and inner ear, as demonstrated in our patient population.

Our work expands upon what has been demonstrated with regards to RT and hearing loss. Prior studies have shown that RT doses to the cochlea greater than 32 Gy are ototoxic when present with CSF shunts, while RT doses greater than 40–45 Gy are independently ototoxic [6,12]. The current Children’s Oncology Group guidelines recommend annual audiology testing for five years after treatment, and every five years afterwards for survivors who received RT dose greater than 30 Gy to the ear, brain, infratemporal or nasopharyngeal field. A one-time audiogram is recommended at least two years after treatment for RT less than 30 Gy [18,19]. Notably, in our patient population, six patients with RT of less than 30 Gy had severe hearing loss.

Audiologic testing can detect hearing loss that may not be detected subjectively by patients or clinically by health care providers [4,5], while even mild to moderate hearing loss has been associated with impaired speech and language development [23,24]. Certain consonant sounds in the English language are indistinguishable below frequencies greater than 4000 Hz [4]. While high-frequency hearing loss may not be subjectively detected in everyday interactions, it can impede language development and understanding of particular sounds. Audiograms remain the gold standard for detecting high-frequency hearing loss. Based on our findings, routine audiologic monitoring may be warranted after RT, even in the absence of subjective hearing loss, among those with lower dose RT. Furthermore, consideration for repeat testing should be given; five patients had severe hearing loss that presented on subsequent testing.

Our study has several limitations. Only patients seen in the ALTFU survivorship program were included, and a small sample of that population had post-treatment audiograms. Post-treatment audiograms that were performed outside the institution may not have been captured. In addition, the reasons for referral to audiology were not always noted. The incidence of aminoglycoside exposure and otitis media was not consistently documented, and therefore, the effects of these comorbidities are unknown. Treatment dates ranged from 1952 to 2016, and for many patients in this study, cochlear dose was unavailable at the time of their treatment. However, all radiation fields where the cochlea may have been inadvertently targeted were included. Further, in 47 of 51 patients with follow-up, there were no indications in clinical notes or audiology reports that hearing loss preceded treatment. Pre-treatment hearing loss may have been present due to congenital choleastoma in two patients, pre-radiation ear surgery in one patient, and acoustic neuroma associated with neurofibromatosis in another patient. Even so, we have included these results in our findings, as these types of patients may be seen in the survivorship setting. Further, we presume in our analysis that normal tympanograms can exclude conductive hearing loss due to middle ear fluid and negative pressure, but these findings do not preclude ossicular conductive hearing loss. Thus, there may be a greater component of conductive hearing loss than identified using tympanograms. Finally, intervals at which audiometric tests were performed were inconsistent and duration of follow-up varied. Thus, it is possible that hearing loss may have been detected earlier if more routine testing was performed.

In contrast to treatment related hearing loss in adult survivors of childhood cancer, aging and noise exposure are the most common causes of hearing loss in the general population. No noise exposures were documented in clinical reports for the patient population. Additionally, all patients but one received their first post-treatment audiogram between ages 4 and 35 years, and 18 of 19 patients who presented with severe hearing loss did so before 40 years of age. Therefore, we conclude that age-related sensorineural hearing loss was not a factor for 18 of the 19 patients with severe impairment.

Ten patients in our study population used hearing aids, and all of these patients exhibited severe impairment on audiogram; other patients with severe impairment on audiogram may also benefit from hearing aids, although an audiological consultation may be needed to make that determination [5]. Barriers, such as stigma, may preclude use of aids. In addition, insurance programs do not typically reimburse the cost of hearing aids or routine audiograms. Several appointments are required to fit the hearing aid, and the average price of a hearing aid can be thousands of dollars. Further research is needed to address this disparity and to determine the accessibility and efficacy of interventions for hearing loss in this population.

Hearing loss presents unique challenges to adult survivors of childhood cancer. Hearing loss has been associated with decreased academic performance, lower prevalence of independent living and employment, and decreased psychosocial outcomes in cancer survivors during critical adolescent and young adult years [25,26,27]. Therefore, detection and intervention for hearing loss is paramount to prevent the negative impact hearing loss can have on quality of life. At the same time, hearing aid use was inconsistent, even among those with severe hearing loss.

In conclusion, our study supports the use of audiologic testing in long-term follow-up for patients with CNS and non-CNS tumors who received RT to fields involving the cochlea. We support the guidelines for audiologic monitoring after high-dose RT, but suggest that routine monitoring may be warranted at RT doses less than 30 Gy and at more routine intervals. Additionally, in light of the social consequences of impaired hearing, further research is needed to determine outcomes for hearing impaired patients and whether those who may benefit from hearing aids have access to them.

## Figures and Tables

**Table 1:** Ototoxicity grading scales. Chang Scale.

Chang		
Grade 0	≤20 dB at 1, 2, and 4 kHz	Normal
Grade 1a	≥40 dB at any freq 6–12 kHz	Normal
Grade 1b	>20 and <40 dB at 4 kHz	Normal
Grade 2a	≥ 40 dB at 4 kHz and above	Normal
Grade 2b	>20 and <40 dB at any freq below 4 kHz	Hearing loss
Grade 3	≥40 dB at 2 or 3 kHz and above	Hearing loss
Grade 4	≥40 dB at 1 kHz and above	Hearing loss

**Table 2:** Ototoxicity grading scales. SIOP Scale.

SIOP		
Grade 0	≤20 dB HL at all frequencies	Normal
Grade 1	>20 dB HL (i.e., 25 dB HL or greater) SNHL above 4000 Hz (i.e., 6 or 8 kHz)	Normal
Grade 2	>20 dB HL SNHL at 4000 Hz and above	Normal
Grade 3	>20 dB HL SNHL at 2000 or 3000 Hz and above	Hearing loss
Grade 4	>40 dB HL (i.e., 45 dB HL or more) SNHL at 2000 Hz and above	Hearing loss

SIOP, International Society of Paediatric Oncology; HL, hearing loss; SNHL, sensorineural hearing loss.

**Table 3:** Demographic characteristics, diagnosis, and treatment exposures for 276 adult survivors of childhood cancer treated with radiation therapy that included the ear.

Characteristics	All Survivors	Audiology	Severe Hearing Loss
	*N* = 276	*n* = 51	*n* = 19
Age at first diagnosis (years)			
Median	10.1	8.4	8.0
Range	0–21.8	0–21.1	0–19.2
Sex (number, %)			
Male	163 (59.1)	27 (52.9)	11 (57.9)
Female	113 (40.9)	24 (47.1)	8 (42.1)
Ethnicity (number, %)			
White	8 (2.9)	37 (72.5)	10 (52.6)
Hispanic	215 (77.9)	3 (5.9)	2 (10.5)
African American	19 (6.9)	3 (5.9)	2 (10.5)
Asian	18(6.5)	5 (9.8)	3 (15.8)
Did not identify or other	16 (5.8)	3 (5.9)	2 (10.5)
Diagnosis (number, %)			
Leukemia	154 (55.8)	22 (43.1)	7 (36.8)
Lymphoma	19 (6.9)	2 (3.9)	0
Rhabdomyosarcoma of head and neck	22 (8.0)	6 (11.8)	2 (10.5)
Retinoblastoma	18 (6.5)	3 (5.9)	2 (10.5)
Other sarcoma	9 (3.2)	2 (3.9)	0
Nasopharyngeal carcinoma	2 (0.7)	2 (3.9)	0
Medulloblastoma	10 (3.6)	4 (7.8)	2 (10.5)
Astrocytoma	11(3.9)	3 (5.9)	0
Ependymoma	2 (3.9)	1 (2.0)	1 (5.3)
CNS germ cell tumor	5 (1.8)	1 (2.0)	0
Other CNS tumors	5 (1.8)	4 (7.8)	5 (26.3)
Other	20 (7.2)	1 (2.0)	0
Follow up in ALTFU (years)			
Median	3.0	4.3	5.3
Range	0–11.1	0–10.1	0–9.9
Audiology follow-up (years)			
Median		15.1	16.6
Range		0–55.1	0–55.1
Age at first post-treatment audiogram (years)			
Median	N/A	19.4	20.0
Range	N/A	7.2–55.1	8.3–55.1
Time from diagnosis to first post-treatment audiogram (years)			
Median	N/A	7.9	9.1
Range	N/A	0.7–55.1	0.7–55.1
Hearing aids (Number, %)	13 (4.7)	10(19.6)	10 (52.6)
Hematopoietic cell transplant	108 (39.1)	1 (2.0)	0
CSF shunt	18(16.5)	3 (5.9)	0
Cranial surgery	42 (15.2)	13 (25.5)	8 (42.1)

ALTFU: Adult Long-Term Follow-Up; CNS: central nervous system; CSF: craniospinal fluid; N/A: not applicable.

**Table 4:** Dose and radiation field for 276 adult survivors of childhood cancer at risk for hearing impairment.

Radiation Field and Dosage	All Survivors	Audiology	Severe Hearing Loss
	*N* = 276	*n* = 51	*n* = 19
All fields			
Median (Gy)	1800	2400	1800
Range (Gy)	200–7200	360–7200	600–7200
Brain (number)	111	13	6
Median (Gy)	1800	2400	3600
Range (Gy)	360–7200	360–7200	600–7200
Total body irradiation (number)	85	16	4
Median (Gy)	1375	1375	1437.5
Range (Gy)	200–2400	450–1500	1350–1500
Orbit (number)	28	6	3
Median (Gy)	4125	3625	4250
Range (Gy)	1000–5949	1000–4500	3000–4250
Nasopharynx (number)	16	6	1
Median (Gy)	5040	5670	N/A
Range (Gy)	900–7200	5040–7200	N/A
Cranial and craniospinal (number)	11	4	3
Median (Gy)	2400	3600	3600
Range (Gy)	600–3600	3500–3600	3500–3600
Sinus (number)	3	2	2
Median (Gy)	5000	5470	6300
Range (Gy)	1200–5940	5000–5940	5400–7200
Skull (number)	4	0	0
Median (Gy)	4280		
Range (Gy)	2100–5580		
Face (number)	6	0	0
Median (Gy)	5295		
Range (Gy)	3000–6300		
Posterior fossa (number)	8	2	0
Median (Gy)	5200	6300	
Range (Gy)	1500–7200	5400–7200	
Pineal gland (number)	2	1	0
Median (Gy)	2610	2160	
Range (Gy)	2160–3060		
More than one field (number)	117	15	6

N/A: not applicable; Gy: Grey.
